# Distribution incidence, mortality of tuberculosis and human development index in Iran: estimates from the global burden of disease study 2019

**DOI:** 10.1186/s12889-023-17114-4

**Published:** 2023-12-04

**Authors:** Hossien Fallahzadeh, Zaher Khazaei, Moslem Lari Najafi, Sajjad Rahimi Pordanjani, Elham Goodarzi

**Affiliations:** 1 Center for Healthcare Data Modeling, Departments of Biostatistics and Epidemiology, School of Public Health, Shahid Sadoughi University of Medical Sciences, Yazd, Iran; 2 Pharmaceutical Sciences and Cosmetic Products Research Center, Kerman University of Medical Sciences, Kerman, Iran; 3 Social Determinants of Health Research Center, Semnan University of Medical Sciences, Semnan, Iran; 4 Social Determinants of Health Research Center, Lorestan University of Medical Sciences, Khorramabad, Iran

**Keywords:** Death, Incidence, Tuberculosis, HDI, Iran

## Abstract

**Background:**

Tuberculosis is one of the most serious challenges facing the global healthcare system. This study aims to investigate the incidence and mortality of tuberculosis in Iran from 2010 to 2019 as well as its relationship with the human development index (HDI).

**Methods:**

The present study is an ecological study aiming at investigating the incidence and mortality of tuberculosis in Iran during the years 2010 to 2019. The related data were extracted from the Global Burden of Disease (GBD) website. The spatial pattern attributed to tuberculosis in the provinces of Iran was analyzed using ArcGIS software. In this study, the two-variable correlation method was used to analyze the data extracted to study the correlation between Tuberculosis and HDI.

**Result:**

Based on the results recorded in GBD, the incidence of tuberculosis in 2010, that is, 14.61 (12.72, 16.74), declined compared to 2019, namely 12.29 (10.71, 14.09). The age-standardized mortality rate which was 1.63 (1.52, 1.73) in 2010, has decreased compared to 2019: 1.17 (1.07, 1.32). The incidence and mortality rates of tuberculosis in Iran in all age groups have decreased in 2019 compared to 2010. The highest incidence and mortality among tuberculosis patients were recorded in Sistan and Baluchistan and Golestan provinces. The results indicated that there was a negative and significant correlation between the mortality rate of tuberculosis and the human development index in 2010 (*r* = -0.509, *P*-value = 0.003) and 2019 (*r* = -0.36, *P*-value = 0.001); however, this correlation between incidence and human development index was not significant (*p* > 0.05).

**Conclusion:**

Since mortality is mostly observed in areas with low HDI, health system policymakers must pay more attention to these areas in order to improve care and perform screenings to diagnose and treat patients thus reducing the mortality rate of tuberculosis and preventing an increase in its incidence in Iran.

**Supplementary Information:**

The online version contains supplementary material available at 10.1186/s12889-023-17114-4.

## Introduction

Policymakers and researchers are paying more and more attention to the significance of infectious diseases for public health and the economic development of countries in the last few decades [1, 2]. Despite its long history of about 3000 years, tuberculosis (TB) is still an acute health problem in human societies [3]. This disease is a global emergency leading to high morbidity and mortality, especially in sub-Saharan African countries, and, after acquired immunodeficiency syndrome (HIV/AIDS), it is considered the second leading fatal infectious disease [4, 5]. Recent estimates of the global burden of people infected with Mycobacterium tuberculosis indicate that about 1.7 billion people, i.e., 23% of the world's population, have this infection. This large number of people with latent tuberculosis is the bed of infectious tuberculosis patients in the future, and despite BCG immunization, adult forms of tuberculosis continue to emerge, indicating that the current vaccine has limited efficacy against adult tuberculosis, hence the need for tuberculosis vaccines with high protective efficiency [1, 4, 6]. It is estimated that by 2030 and 2050, latent tuberculosis patients will generate 16.3 and 8.3 active tuberculosis patients per 100,000 population, respectively [7]. The incidence of tuberculosis varies considerably among different countries and in different population groups and within countries [8, 9].

The incidence rate of tuberculosis is about 365 cases per 100,000 people in Africa; it is about 5 people per 100,000 people in London; it is about 21 people per 100,000 people in Spain; it is 5 people per 100,000 people in America; and globally, on average, it is 13 cases per 100,000 people. In Iran, the incidence rate of tuberculosis reached 16 cases per 100,000 people in 2015 and declined to 14 cases per 100,000 people in 2016 and 2017, and fell to 13 cases per 100,000 people in 2020, where 4% of cases of tuberculosis patients were under 14 years old [5, 10, 11].

Iran’s wide borders with tuberculosis-affected countries such as Azerbaijan, Turkmenistan, Armenia, Pakistan, Afghanistan, and Iraq lead to an increase in immigration and travel to Iran from neighboring countries, which seriously impedes tuberculosis control. Since the distribution of the disease is different in different regions of the country, effective screening programs are needed to identify high-risk areas [12, 13]. Health disparities refer to differences in the incidence, prevalence, mortality, burden of disease, and other adverse health conditions experienced by certain population groups. A comprehensive literature review of other articles indicated that in every country, there is a disproportionately higher prevalence of communicable diseases among vulnerable groups, i.e., those with low levels of education or income, immigrants, and people with high-risk lifestyles [14–16]. Many social, environmental, and biological risk factors of tuberculosis are prevalent among the poor versus the wealthier people [17]. These factors probably contribute to a complex network of risk factors based on the relationship between poverty and tuberculosis [18, 19]. Human Development Index (HDI) is a three-dimensional measure including life expectancy, education, and income. In addition, HDI is one of the most important indices indicating the level of development in each region [20]. The level of the human development index is a strong predictor of the changes in the incidence of tuberculosis over time in the countries [21, 22]. This study aims to investigate the incidence and mortality of tuberculosis in Iran from 2010 to 2019 based on the global burden of disease data and its relationship with the human development index in the provinces of the country.

## Methods

### Data sources

The Institute for Health Metrics and Evaluation (IHME) produces annual updates to the GBD study, including temporal and geographic trends, since 1990. Updating new data and methodological advances to provide policymakers with the most up-to-date information for health care planning and resource allocation. The 2019 GBD study estimated incidence, prevalence, and mortality by age, sex, year, and location for 354 diseases and injuries and 3484 sequelae i.e., disabling consequences of these diseases and injuries [23].

This ecological study in Iran was designed to investigate the distribution of the incidence and mortality of tuberculosis and its relationship with the human development index. All the data used in this research were made available to the public at http://ghdx.healthdata.org/gbd-results-tool. Data were extracted using GBD results. These data including mortality and incidence estimates for all age and sex groups along with the 95% CI were accessible. For some indices, the percentage change between 1990 and 2019 was reported [24].

TB incidence (number of new cases of the disease in the population per 100,000) and mortality (number of deaths per 100,000) were collected by the GBD from various data sources.

The data relating to the human development index was extracted from the United Nations Development Program.

The Human Development Index (HDI) is a statistical tool used to measure a country's overall achievements in its social and economic dimensions. According to this index, the social and economic dimensions of a country are evaluated based on the health of people, their level of education, and their standard of living. The United Nations measures the HDI index annually for the member countries of the United Nations in a report based on which different countries are ranked [25, 26].

### Data analysis

The analysis of the spatial pattern attributed to tuberculosis in the provinces of Iran was done using ArcGIS software.

In this study, the two-variable correlation method was used to analyze the data extracted to study the correlation between Tuberculosis and HDI. The significance level was *P* < 0.05. The analyses were made using Stata software version 12 (Stata Corp, College Station, TX, USA).

## Results

Based on the results recorded in GBD, the incidence of tuberculosis in 2010, that is, 14.61 (12.72, 16.74), declined compared to 2019, namely 12.29 (10.71, 14.09). The age-standardized mortality rate which was 1.63 (1.52, 1.73) in 2010, has decreased compared to 2019: 1.17 (1.07, 1.32). The results showed that the age-standardized prevalence rate of tuberculosis in Iran was 27,251.1 (24,466.9, 30,263.7) in 2010 and 25,365.7 (22,803.1, 28,334.3) in 2019. The results are presented by sex in Table 1.

**Table 1:** Incidence, mortality, and prevalence rate in Iran (source: Global Burden of Disease)

	Age-standardized Rate								
	Male			Female			Both		
	2010	2019	Change 2010–2019	2010	2019	Change 2010–2019	2010	2019	Change 2010–2019
Incidence	14.24 (12.39, 16.4)	13.49 (11.69, 15.5)	-0.05 (-0.09, -0.008)	15.01 (12.97, 17.37)	11.07 (9.57, 12.76)	-0.26 (-0.29, -0.23)	14.61 (12.72, 16.74)	12.29 (10.71, 14.09)	-0.15 (-0.18, -0.12)
Death	2.02 (1.87, 2.15)	1.34 (1.23, 1.45)	-0.33 (-0.38, -0.27)	1.23 (1.11, 1.34)	0.99 (0.87, 1.28)	-0.19 (-0.28, 0.11)	1.63 (1.52, 1.73)	1.17 (1.07, 1.32)	-0.28 (-0.33, -0.17)
Prevalence	26,687.9 (23,917.4, 29,672.8)	24,618.09 (22,096.8, 27,590.7)	-0.07 (-0.1, -0.04)	27,825.8 (24,946.6, 30,910.2)	26,113.9 (23,530.4, 29,059.3)	-0.06 (-0.08, -0.03)	27,251.1 (24,466.9, 30,263.7)	25,364.7 (22,803.1, 28,334.3)	-0.06 (-0.09, -0.04)

**Fig. 1:**
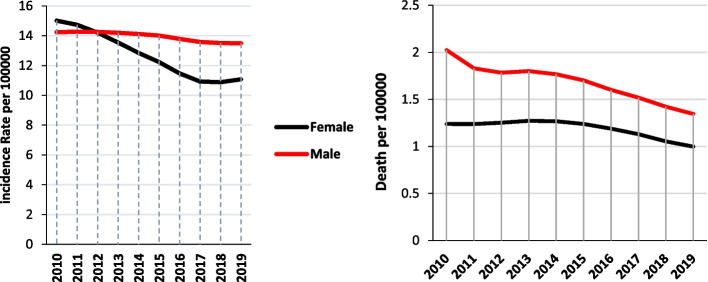
The incidence and mortality trend of tuberculosis patients in Iran during 2010–2019 based on sex (source: Global Burden of Disease)

The results showed that in 2010, the incidence rate in women was higher than that in men, and during the following years, this incidence in women decreased and became less than in men. Mortality decline has occurred among men from 2010 to 2019, and in women, although the trend of mortality has been almost constant between 2010 and 2014, since 2014, there has been a decreasing trend (Fig. 1).

**Fig. 2:**
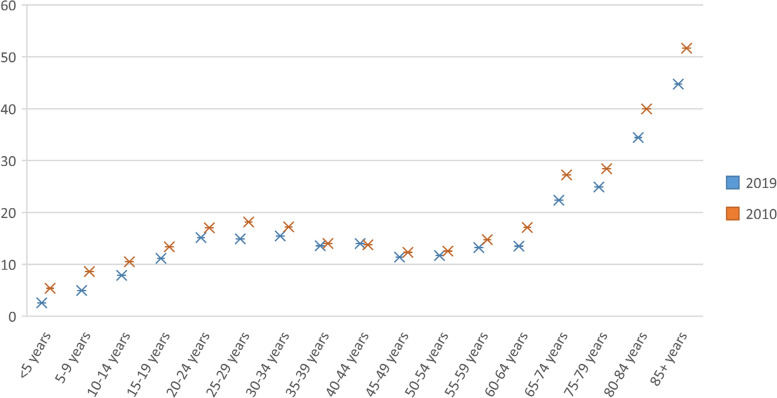
Comparing the incidence rate of Tuberculosis in 2010 VS 2019 by age group in Iran (source: Global Burden of Disease)

Figure 2 compares the incidence rate of tuberculosis in 2010 compared to 2019 based on age groups. It can be observed that the incidence rate of tuberculosis has declined in all age groups in 2019 compared to 2010, and the highest incidence rate of tuberculosis can be seen among age groups above 60 years and the lowest incidence rate of tuberculosis is related to children under 5 years old.

**Fig. 3:**
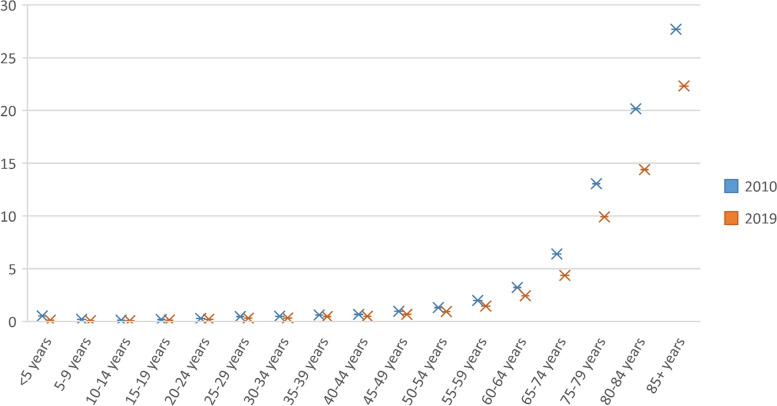
Comparing mortality rate of Tuberculosis in 2010 VS 2019 by age group in Iran (source: Global Burden of Disease)

Figure 3 compares the mortality rate of tuberculosis in 2010 compared to 2019 based on age groups. As can be seen in all age groups, the mortality rate of tuberculosis has decreased in 2019 compared to 2010, and the highest incidence rate of Tuberculosis is seen among the age groups above 60 years old, and a sharp decline in the mortality rate is observed in these age groups.

**Fig. 4:**
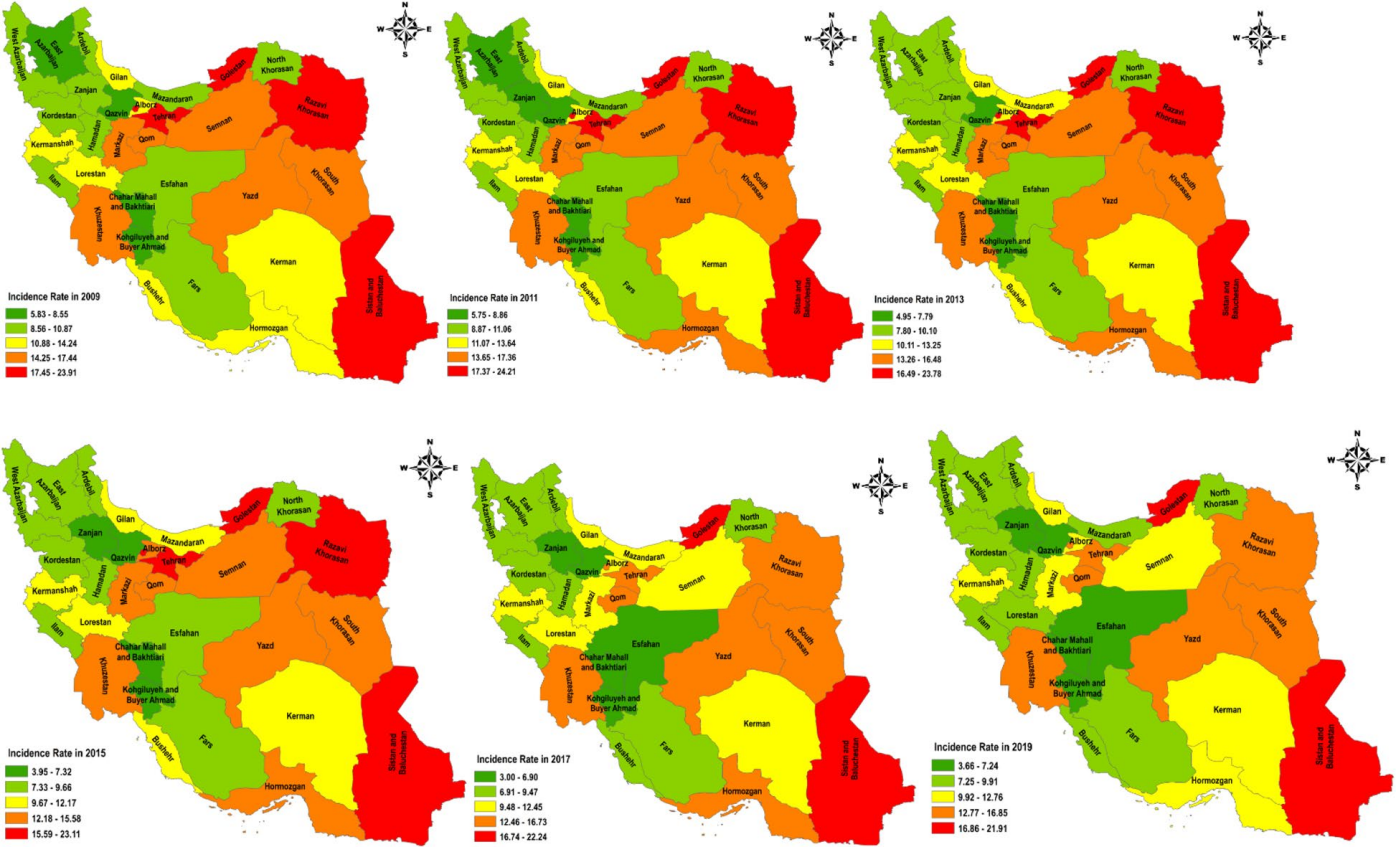
Distribution of Incidence Rate of Tuberculosis in Iran During 2009–2019 (source: Global Burden of Disease)

Figure 4 shows the incidence rate of tuberculosis in different provinces of the country from 2009 to 2019. As can be seen, the provinces of Sistan and Baluchistan, Khorasan Razavi, Golestan, and Tehran had the highest incidence rate in the country until 2015. In 2017 and 2019, the incidence rate decreased in Tehran and Khorasan Razavi provinces, but Sistan and Baluchestan and Golestan provinces still had the highest incidence rate of tuberculosis (Fig. 3).

**Fig. 5:**
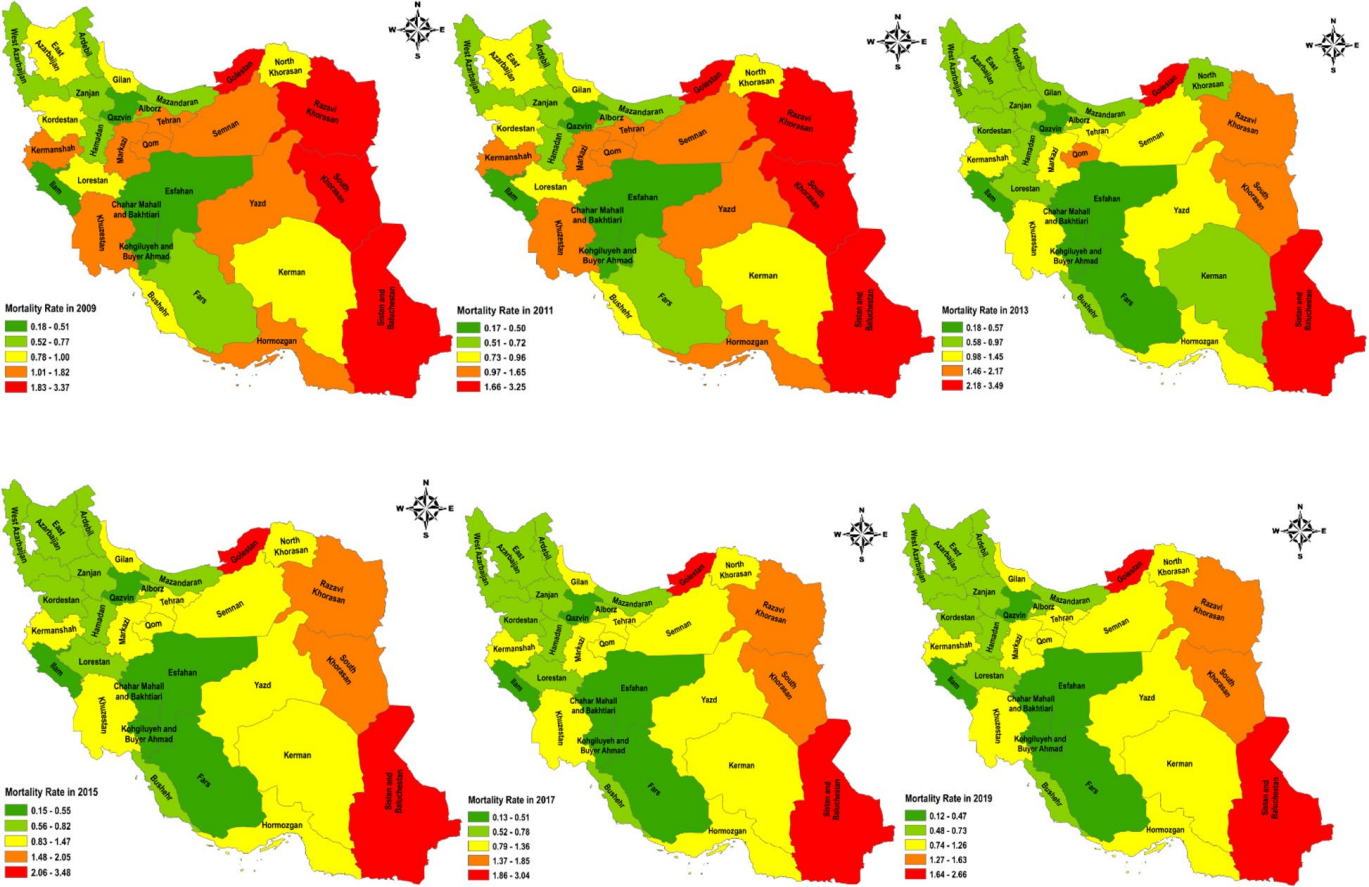
Distribution of death rate of Tuberculosis in Iran from 2009 to 2019 (source: Global Burden of Disease)

Figure 5 shows the mortality rate of tuberculosis in different provinces of Iran from 2009 to 2019. As can be seen, the provinces of Sistan and Baluchistan, Khorasan Razavi, Golestan, and South Khorasan had the highest mortality rate in the country until 2011. And in 2015 to 2019, the highest mortality rate from tuberculosis was seen in Golestan, and Sistan and Baluchistan provinces (Fig. 5).

**Fig. 6:**
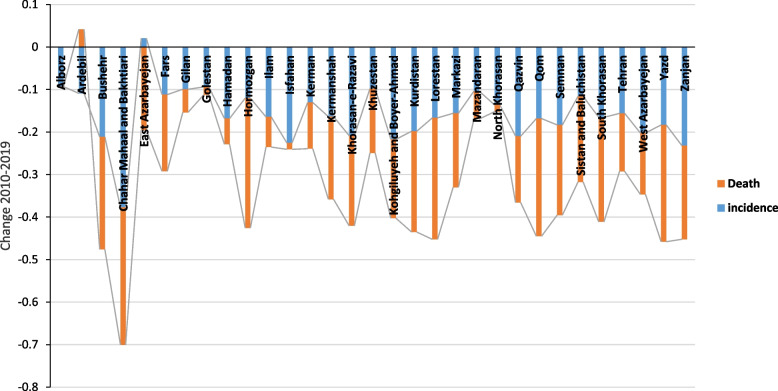
Comparing the change in incidence and death in 2010–2019 in Iran (source: Global Burden of Disease)

Figure 6 shows the changes in incidence and death in Iran by province. It can be seen that in all provinces of the country (except Ardabil province), the changes in 2010–2019 related to incidence and death were negative, which indicates a decrease in the incidence rate and death rate during these years.

Table 2 provides a comparison of the incidence rate of tuberculosis in women and men by the province in 2010 compared to 2019 and it also compares the changes from 2010 to 2019 in the provinces. It can be seen that the incidence rate in women shows a negative change in all provinces in 2019 compared to the year 2010, which indicates a decline in the incidence rate in women. However, the rate of changes in incidence among men has been positive in some provinces of the country, including Alborz, Ardabil, West Azerbaijan, Fars, Gilan, Hormozgan, Mazandaran, Qom, Sistan and Baluchistan, Kerman, Khuzestan, and Tehran, which indicates an increase in incidence rate among men in these provinces (Table 2).

**Table 2:**
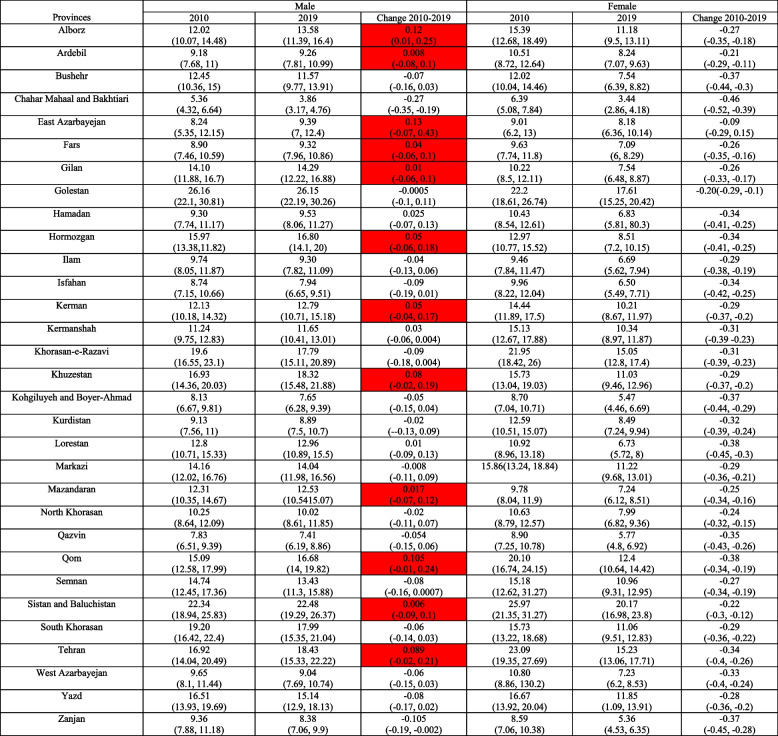
The incidence rate of Tuberculosis in 2010 VS 2019 by sex in Iran (All ages) (source: Global Burden of Disease)

Table 3 compares the death rate of tuberculosis by province in men and women in 2010 compared to 2019 and shows the changes from 2010 to 2019 in all provinces. It can be seen that the death rate in men and women shows negative changes in 2019 compared to 2010 in all provinces, indicating a decrease in the death rate in men and women (Table 3).

**Table 3:** The death rate of Tuberculosis in 2010 VS 2019 by sex in Iran (All ages) (source: Global Burden of Disease)

Death	Male			Female		
	2010	2019	Change 2010–2019	2010	2019	Change 2010–2019
Alborz	0.858 (0.72, 1)	0.824 (0.64, 1.04)	-0.039 (-0.25, 0.23)	0.607 (0.5, 0.7)	0.638 (0.5, 0.82)	0.051 (-0.2, 0.46)
Ardebil	0.796 (0.69, 0.92)	0.769 (0.64, 0.92)	-0.034 (-0.21, 0.19)	0.513 (0.44, 0.58)	0.594 (0.48, 0.78)	0.157 (-0.07, 0.6)
Bushehr	1.05 (0.95, 1.15)	0.739 (0.63, 0.85)	-0.297 (-0.4, -0.17)	0.539 (0.47, 0.6)	0.446 (0.37, 0.6)	-0.171 (-0.32, 0.15)
Chahar Mahaal and Bakhtiari	0.224 (0.18, 0.26)	0.149 (0.12, 0.18)	-0.331 (-0.47, -0.05)	0.123 (0.1, 0.14)	0.084 (0.06, 0.12)	-0.314 (-0.5, 0.06)
East Azarbayejan	1.03 (0.18, 0.26)	0.754 (0.61, 0.93)	-0.267 (-0.42, -0.05)	0.571 (0.49, 0.66)	0.535 (0.41, 0.78)	-0.063 (-0.29, 0.44)
Fars	0.756 (0.64, 0.87)	0.589 (0.46, 0.73)	-0.22 (-0.39, -0.01)	0.37 (0.31, 0.43)	0.331 (0.26, 0.46)	-0.103 (-0.31, 0.28)
Gilan	1.39 (1.21, 1.61)	1.22 (0.99, 1.49)	-0.118 (-0.3, 0.1)	0.545 (0.47, 0.62)	0.597 (0.47, 0.79)	0.094 (-0.15, 0.46)
Golestan	3.41 (3.01, 3.81)	3.11 (2.57, 3.74)	-0.085 (-0.26, 0.11)	1.56 (1.37, 1.75)	1.75 (1.41, 2.33)	0.12 (-0.11, 0.53)
Hamadan	0.885 (0.75, 1.03)	0.768 (0.61, 0.93)	-0.132 (-0.31, 0.09)	0.459 (0.39, 0.52)	0.493 (0.39, 0.65)	0.074 (-0.15, 0.52)
Hormozgan	1.93 (1.69, 2.2)	1.27 (1.04, 1.51)	-0.343 (-0.46, -0.19)	0.781 (0.68, 0.88)	0.618 (0.49, 0.88)	-0.209 (-0.37, 0.18)
Ilam	0.674 (0.45, 0.62)	0.572 (0.48, 0.68)	-0.151 (-0.3, 0.02)	0.322 (0.28, 0.36)	0.354 (0.28, 0.48)	0.098 (-0.12, 0.55)
Isfahan	0.536 (0.45, 0.62)	0.488 (0.48, 0.68)	-0.088(-0.29, 0.16)	0.359 (0.3, 0.42)	0.395 (0.31, 0.53)	0.099 (-0.16, 0.55)
Kerman	1.25 (1.08, 1.42)	1.02 (0.84, 1.23)	-0.18 (-0.32, 0.01)	0.731 (0.63, 0.83)	0.739 (0.59, 0.98)	0.011 (-0.19, 0.36)
Kermanshah	1.68 (1.45, 1.94)	1.25 (1.01, 1.51)	-0.256 (-0.41, -0.06)	0.9 (0.77, 1.04)	0.832 (0.66, 1.14)	-0.075 (-0.28, 0.34)
Khorasan-e-Razavi	2.53 (2.24, 2.84)	1.82 (1.51, 2.17)	-0.279 (-0.41, -0.11)	1.59 (1.37, 1.82)	1.42 (1.12, 2)	-0.106 (-0.31, 0.34)
Khuzestan	1.94 (1.7, 2.2)	1.53 (1.25, 1.86)	-0.211(-0.37, -0.01)	0.835 (0.72, 0.94)	0.826 (0.64,1.17)	-0.011 (-0.24, 0.42)
Kohgiluyeh and Boyer-Ahmad	0.51 (0.41, 0.61)	0.405 (0.31, 0.51)	-0.206 (-0.41, 0.07)	0.195 (0.15, 0.23)	0.164 (0.12, 0.23)	-0.158 (-0.39, 0.31)
Kurdistan	0.94 (0.81, 1.08)	0.645 (0.52, 0.78)	-0.313 (-0.45, -0.14)	0.70 (0.6, 0.81)	0.60 (0.48, 0.84)	-0.134 (-0.33, 0.25)
Lorestan	1.39 (1.17, 1.64)	0.953 (0.77, 1.17)	-0.314 (-0.45, -0.1)	0.521 (0.44, 0.6)	0.41 (0.3, 0.64)	-0.213 (-0.42, 0.23)
Markazi	1.7 (1.47, 1.95)	1.24 (1.02, 1.49)	-0.268 (-0.42, -0.08)	0.936 (0.78, 1.08)	0.937 (0.74, 1.25)	0.001 (-0.23, 0.39)
Mazandaran	1.01 (0.86, 1.17)	0.865 (0.71, 1.05)	-0.144 (-0.32, 0.07)	0.38 (0.32, 0.43)	0.435 (0.34, 0.57)	0.144 (-0.11, 0.55)
North Khorasan	1.15 (1.01, 1.32)	1 (0.83, 1.19)	-0.125 (-0.28, 0.06)	0.591 (0.51, 0.67)	0.704 (0.55, 0.96)	0.191 (-0.06, 0.67)
Qazvin	0.67 (0.58, 0.75)	0.542 (0.45, 0.64)	-0.191 (-0.33, -0.01)	0.35 (0.3, 0.4)	0.321 (0.25, 0.44)	-0.090 (-0.29, 0.31)
Qom	1.96 (1.71, 2.23)	1.36 (1.12, 1.65)	-0.302 (-0.43, -0.14)	1.50 (1.3, 1.72)	1.14 (0.91, 1.56)	-0.242 (-0.4, 0.09)
Semnan	1.37 (1.2, 1.5)	1.03 (0.86, 1.21)	-0.244 (-0.37, -0.08)	0.916 (0.79, 1.05)	0.766 (0.59, 1.07)	-0.163 (-0.34, 0.14)
Sistan and Baluchistan	3.99 (3.41, 4.64)	3.02 (2.41, 3.75)	-0.242 (-0.41, -0.01)	2.66 (2.21, 3.12)	2.28 (1.78, 3.09)	-0.141 (-0.34, 0.28)
South Khorasan	2.79 (2.44, 3.18)	1.95 (1.62, 2.32)	-0.301 (-0.42, -0.15)	1.27 (1.1, 1.47)	1.11 (0.89, 1.51)	-0.13 (-0.3, 0.23)
Tehran	1.27 (1.06, 1.52)	1.07 (0.85, 1.35)	-0.162 (-0.36, 0.01)	1.14 (0.91, 1.38)	1.02 (0.8, 1.31)	-0.106 (-0.32, 0.25)
West Azarbayejan	0.914 (0.8, 1.03)	0.706 (0.58, 0.85)	-0.227 (-0.37, -0.06)	0.45 (0.4, 0.52)	0.469 (0.37, 0.67)	0.0216 (-021, 0.53)
Yazd	1.71 (1.45, 1.99)	1.15 (0.93, 1.4)	-0.325 (-0.46, -0.15)	0.98 (0.84, 1.15)	0.811 (0.62, 1.16)	-0.178 (-0.37, 0.24)
Zanjan	1.03 (0.91, 1.16)	0.734 (0.61, 0.86)	-0.289 (-0.4, -0.16)	0.34 (0.29, 0.38)	0.334 (0.26, 0.48)	-0.025 (-0.25, 0.43)

**Fig. 7:**
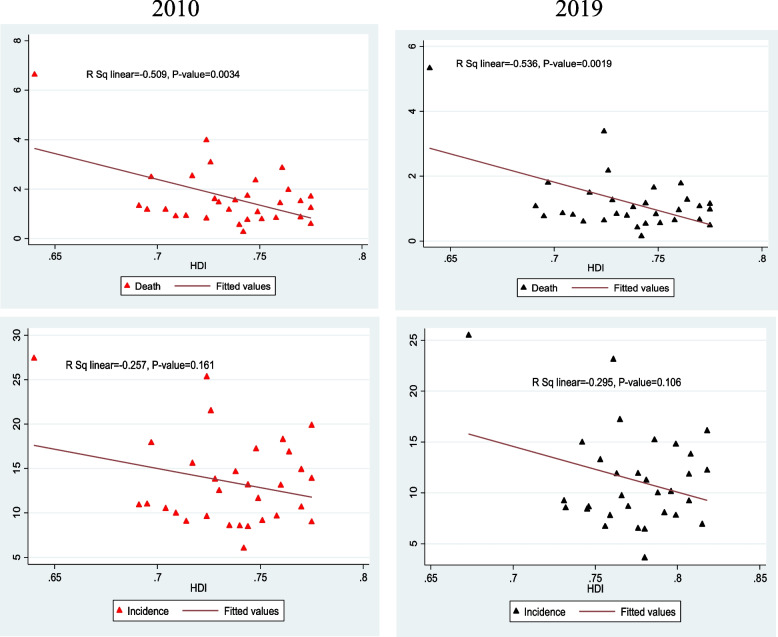
Correlation between the Human Development Index, incidence and Death rates of Tuberculosis in Iran in 2010 and 2019

The results showed that there was a negative and significant correlation between the death rate of tuberculosis and the human development index in the years 2010 and 2019. Considering the relationship between the incidence rate and the HDI index, the results showed that there was a negative correlation between the incidence rate and the HDI index in the years 2010 and 2019, but this correlation was not statistically significant (Fig. 7).

## Discussion

Tuberculosis cases occur in every part of the world. In 2021, the largest number of new cases of TB was found in Southeast Asia with 46% of new cases, followed by Africa with 23% of new cases, and the Western Pacific with 18%. Globally, the incidence of tuberculosis is falling, but not fast enough to reach the 2020 milestone of a 20% reduction between 2015 and 2020 [27]. Glaziou et al. reported a decreasing trend in the incidence, prevalence, and mortality rate of tuberculosis in the world between 1990 and 2015. According to the United States CDC report, the incidence rate of tuberculosis has been decreasing during 1993–2010 [28].

One of the key factors in healthcare planning in any society is determining the incidence of diseases. Knowing about the pattern of changes in the incidence of diseases in a country can be of paramount importance for planning strategies at the country level. Public health organizations argue that assessment or surveillance of disease incidence trends, mortality rate, and disease risk factors might contribute to the occurrence of adverse health events [29]. In Iran, important measures have been taken to control tuberculosis, leading to some achievements. The present study showed that the incidence and death rates of tuberculosis in Iran in all age groups declined in 2019 compared to 2010.

Based on the Iranian Ministry of Health reports, the total incidence of tuberculosis in Iran decreased by 1.17% between 2005 and 2015, while it increased by approximately 4% in Mazandaran province [27]. A study conducted in the west of Iran (Kurdistan province) showed that the incidence rate has been decreasing during the years 2000–2012 [30]. A study on Iran conducted during 1998–2009 showed a decreasing trend [31]. Another study in Iran pointed to a decreasing trend in the incidence of tuberculosis from 1995 to 2012 [32]. Noeske et al., in a study in Cameroon, reported a decrease in PTB + cases from 139 to 121 cases per 1000 people during 2006–2014 [33].

The risk of contracting tuberculosis is higher among the elderly population in the world. According to the present study, the highest incidence and death rate of tuberculosis is seen in the age groups above 60 years and the lowest rate is related to children under 5 years old, which may be the effect of reducing immunity in the lungs in the elderly and shows the significant success of the country in controlling the disease in younger age groups. In the study by Khajedaluee et al. (2019), the mortality rate was higher in the elderly group [34]. Hagiya et al. (2019) investigated the incidence of active tuberculosis and mortality among the elderly and showed that although decreasing steadily, the incidence and death rates are still high [35].

According to the present study, the incidence and death due to tuberculosis during the years 2010 to 2019 were higher in men, and the decreasing trend of the disease in women was more significant than in men. Based on the documents, men get lower health services than women [31, 36]. Male patients with tuberculosis usually postpone health care more than female patients [30].

Although all parts of Iran are under the surveillance of the comprehensive tuberculosis control program, the incidence of tuberculosis is not the same in all regions of the country. According to the prior studies, although Golestan province is not bordering provinces with a high prevalence of tuberculosis, in 2005, it ranked second in terms of incidence and prevalence of tuberculosis with a rate of 38.1 per 100,000. Sistan and Baluchistan ranked first in overall TB prevalence with a rate of 44.1 per 100,000 people. While Mazandaran province, Golestan province’s neighbor, with a similar ecosystem, has a tuberculosis incidence of 9.6 per 100,000, which is much lower [37].

This study showed that Sistan and Baluchestan and Golestan provinces had the highest incidence and death rates of tuberculosis. Due to its proximity to Afghanistan and Pakistan, the province of Sistan and Baluchistan had a high prevalence of tuberculosis. The results of the study conducted by Salek et al. in Golestan province showed that the incidence rate of smear-positive pulmonary tuberculosis in Golestan and the national incidence rate in the same year were 22.1 and 7.8 per 100,000 people, respectively [37]. The high rate of tuberculosis in this province can be due to being located on the border.

The results of the present study showed that there was a negative and significant correlation between the death rate of tuberculosis disease and the human development index in 2010 and 2019. The results of Okhovat-Isfahani et al. [17], Muniyandi et al. [38], and Rodríguez-Morales et al. [39] showed a higher concentration of TB in countries with low HDI. Inequality in TB incidence has been observed based on WHO regions. In most WHO regions, tuberculosis and TB/HIV were concentrated in countries with lower HDI [17]. According to some studies, poverty, income inequality and lack of social capital were important predictors of an increase in tuberculosis incidence [21, 40, 41].

The reasons behind the higher concentration of TB incidence in less developed areas may be living in poor conditions such as food insecurity, inadequate housing, and lack of access to proper health care [42]. Therefore, in areas with lower HDI, a control program with a public health approach is needed. In fact, every effort should be made to improve social conditions, including reducing the incidence and death of infectious diseases in these areas.

One of the limitations of this study was that since the current study was an ecological study, the most important error in this study was an ecological fallacy, and the results of these studies should be interpreted with caution. Furthermore, other limitations to be mentioned here are those reported in GBD studies and the lack of accurate and reliable data for the incidence and mortality rates in some provinces of Iran, especially in the more deprived areas.

## Conclusion

Studying the trend of changes in the prevalence or incidence rates provides valuable information for assessing the needs, and designing and revising development plans and indices in a country. Evaluation of data related to one period can also help predict the frequency of future incidents. To achieve the objectives of TB control, activities leading to timely detection and effective treatment of patients in each country and province should be included in TB control strategies. On the other hand, based on the results of mortality in 2010, mortality rates were higher in areas with lower HDI, and this relationship and correlation were also observed in 2019, showing that despite the passage of several years and a decrease in the incidence and death rates of tuberculosis, in Areas with a lower human development index, more deaths occur due to tuberculosis, which shows that these areas should be included in the priorities and planning of the livelihood policy so that they can be effective in reducing deaths resulting from this disease in the country.

### Supplementary Information

**Additional file 1.**

## Data Availability

All the data used in this research were made available to the public at http://ghdx.healthdata.org/gbd-results-tool
