# Correction: Distribution incidence, mortality of tuberculosis and human development index in Iran: estimates from the global burden of disease study 2019

**DOI:** 10.1186/s12889-024-18021-y

**Published:** 2024-03-01

**Authors:** Hossien Fallahzadeh, Zaher Khazaei, Moslem Lari Najafi, Sajjad Rahimi Pordanjani, Elham Goodarzi

**Affiliations:** 1 Center for Healthcare Data Modeling, Departments of Biostatistics and Epidemiology, School of Public Health, Shahid Sadoughi University of Medical Sciences, Yazd, Iran; 2 Pharmaceutical Sciences and Cosmetic Products Research Center, Kerman University of Medical Sciences, Kerman, Iran; 3 Social Determinants of Health Research Center, Semnan University of Medical Sciences, Semnan, Iran; 4 Social Determinants of Health Research Center, Lorestan University of Medical Sciences, Khorramabad, Iran

**Correction: BMC Public Health (2023) 23:2404**

10.1186/s12889-023-17114-4

The original publication of this article contained an incorrect Ethics approval and consent to participate. The incorrect and correct information is shown in this correction article, the original article has been updated.

**Incorrect**

The study was approved by the ethics committee of Yazd University of Medical Science, Iran.

**correct**

The study was approved by the ethics committee of Yazd University of Medical Science, Iran. IR.SSU.SPH.REC.1401.202

