# Supplementary material for: Distribution incidence, mortality of tuberculosis and human development index in Iran: estimates from the global burden of disease study 2019
Source: BMC Public Health. 2023 Dec 4;23:2404. doi: 10.1186/s12889-023-17114-4 (PMC10694928; doi:10.1186/s12889-023-17114-4)
Supplement: Supplementary file 1 — Additional file 1. [file 12889_2023_17114_MOESM1_ESM.doc]

1. What is known?

Tuberculosis (TB) is leading cause of more than 1 million deaths worldwide each year which most of them occur in low- and middle-income countries. Determining trends in incidence, prevalence, and mortality of TB is a critical issue to evaluate the success of programs for TB control. Programming and identification of challenges are essential for care and prevention of TB. More attention to it in Iran is necessary, because Iran's neighborhoods, Afghanistan and Pakistan, are among the 22 countries of the world in which TB is prevalent. According to Communicable Disease Center of Iran predicted that the rates of case finding and successful treatment of TB will increase to more than 85 and 90%, respectively.

1. What does the study add?

Determining trend of incidence, prevalence, and mortality of TB is essential for assessing the success of TB control programs and identifying remaining challenges. This study provides a comprehensive assessment of the incidence, mortality and burden of tuberculosis. In addition, it includes the analysis of the relationship between the burden of tuberculosis and the human development index (this index evaluates social and economic dimensions of a country based on level of individual health, education and their standard of living.). To determine fundamental areas for prioritizing resources for further research and intervention by assessing the distribution of tuberculosis in Iran related to its social and economic situation.

1. What are implications for clinical practice, public health and / or research?

While mortality of TB in Iran is decreasing, it is still an important issue. Therefore, strengthening health systems for early detection of TB and improvement in diagnosis, treatment and follow-up should be prioritized. Because of higher incidence, mortality, and burden of tuberculosis in some regions of Iran, efforts should be made to investigate the reasons for the differences and address them.

brief title: Incidence and Mortality of Tuberculosis and Human Development Index in Iran
